# Transitional Pain Service: An Update

**DOI:** 10.1007/s11916-024-01239-1

**Published:** 2024-03-26

**Authors:** Ruben Klimke, Alexander Ott, Carolina S. Romero, Andrea Berendes, Richard D. Urman, Markus M. Luedi, Vighnesh Ashok

**Affiliations:** 1 Department of Anaesthesiology Rescue- and Pain Medicine, Cantonal Hospital of St. Gallen, St. Gallen, Switzerland; 2 Department of Anaesthesiology and Critical Care, Hospital General Universitario de Valencia, Valencia, Spain; 3 Research Methods Department, Universidad Europea de Valencia, Valencia, Spain; 4 Center for Palliative Care Medicine, Cantonal Hospital of St. Gallen, St. Gallen, Switzerland; 5 Department of Anaesthesiology, College of Medicine, The Ohio State University, Columbus, OH 43210 United States of America; 6 Department of Anaesthesiology and Pain Medicine, Inselspital, Bern University Hospital, University of Bern, Bern, Switzerland; 7 Department of Anaesthesia and Intensive Care, Post Graduate Institute of Medical Education and Research, Chandigarh, India

**Keywords:** Chronic pain, Postsurgical pian, Transitional pain service, Perioperative pain, Opioids

## Abstract

**Purpose of Review:**

Chronic Postsurgical Pain (CPSP) and the risk for long-term opioid dependency are known complications following major surgery. The idea of Transitional Pain Service (TPS) has been introduced as an interdisciplinary setting to manage pain in the perioperative continuum. We expand on the basic framework and principles of TPS and summarize the current evidence of the TPS and possible interventions to adress postoperative pain. Areas of future work in TPS-related research are discussed.

**Recent Findings:**

Several studies support the effectiveness of TPS in reducing opioid consumption in the perioperative period and following discharge. Some studies also show an improvement in functional outcome with TPS with patients reporting lower pain severity and pain interference.

**Summary:**

The TPS aims to halt the progress of acute postoperative pain to CPSP by providing longitudinal support with patient-centered care. While some studies suggest a positive impact of TPS implementation in terms of reduction in postoperative opioid consumption and improvement of some functional outcomes, direct evidence in terms of reduction in the incidence of CPSP is still missing. The cost-effectiveness of TPS and the expansion of TPS through e-health services and digital applications also need to be evaluated.

## Introduction

Chronic Postsurgical Pain (CPSP) is defined as “pain that develops or increases in intensity after a surgical procedure or tissue injury, and persists beyond the healing process i.e. at least 3 months after the initiating event” [1•, 2]. While thought to be widely underreported, the incidence of CPSP has shown to vary from 5% up to as high as 85% [1•]. The risk of developing CPSP varies between different surgical procedures, with limb amputations, inguinal hernia repair, spine surgery, thoracotomy and mastectomy associated with higher incidence of CPSP [3, 4]. With the new ICD-11 classification of the CPSP, classification is clearer. CPSP is considered a secondary illness and not just a symptom [1•]. The exact mechanisms of transition from acute postoperative pain to CPSP are still mostly unknown [5]. Biological, psychological, social, and environmental factors have shown to play contributory roles [6•].

The development of CPSP after surgery is a major concern for several reasons. These patients often have limited therapeutic options available, and are frequently prescribed potent opioids at escalating doses and/or for longer duration of time than normally necessary. This increased perioperative opioid consumption in postoperative patients has been linked to not just poorer functional and affective health outcomes, but also with higher morbidity and mortality [7–9]. Furthermore, studies have shown that opioid prescriptions at discharge are present in 49 to 95% of patients, with most of them missing a safe and rational opioid weaning plan [8]. While it is commonly expected that postoperative pain decreases over time with patients tapering their opioid medications following discharge from the healthcare facility, in reality studies have shown that between 3 and 14% of former opioid naive patients continue to use opioids for more than 3 months after hospital discharge [7, 10, 11•]. These findings have important implications in the current context of the opioid epidemic in North America and Europe. Understandably, patients who develop CPSP also use a disproportionate quantity of healthcare resources due to poor postoperative pain management, prolonged hospital stay, and emergency visits or readmissions due to severe pain exacerbations following discharge, resulting in significantly exaggerated healthcare costs [12, 13]. In fact, it has been estimated that CPSP results in an additional annual cost of about 41,000 dollars per patient in terms of healthcare resource utilization in the United States [14•]. In addition, patients with CPSP demonstrate a lower quality of life regardless of opioid use [15].

Therefore, we aim to describe the rationale, principles, and key components of a novel concept of Transitional Pain Service (TPS). We would then go on to summarize the existing evidence base for the utility of the TPS and its perioperative impact on patients undergoing surgery, before providing some key recommendations for further work in the field of TPS and CPSP.

## Transitional Pain Service

In order to better address the occurrence and negative consequences of CPSP, the idea of Transitional Pain Service (TPS) was conceptualized in 2014 [16••]. TPS was introduced as an interdisciplinary service with three primary goals: (1) approach to pre-and postoperative pain management for patients at risk for CPSP or pain disability, (2) opioid management for medically complex patients post-discharge, and (3) improvement of patient coping and functioning to ensure an as high as possible quality of life after surgery 2014 [16••]. It is constructed around the perioperative continuum, but continues to provide care to patients for up to 6 months after discharge. This program supports the long-term multidisciplinary pain management of patients at risk of developing chronic pain while simultaneously focusing on their opioid tapering plan [11•, 17]. As a holistic approach, the TPS aims to form a continuous link between preoperative preparation, acute postoperative pain management in the hospital, and long-term pain management and follow-up after discharge [5, 18].

Typically, a TPS team is interdisciplinary, comprising anesthesiologists, acute and chronic pain specialists, pain nurse practitioners, psychologists, physiotherapists, and patient care coordinators [19, 20••, 21]. There are 3 main interventions provided by the TPS team: (i) optimal acute postoperative pain control using multimodal analgesia and judicious use of opioids with an aim to facilitate reduction of postoperative opioid use; (ii) non-pharmacological interventions based on physiotherapy and yoga; (iii) psychological therapy centered around preoperative patient education and cognitive behavioral therapy based on the acceptance and commitment model of psychotherapy [21, 22].

In this way, TPS aims to address the gap between preoperative, in-hospital, and outpatient care of patients undergoing surgery. The usual fragmentation of postoperative pain management between surgeons, anesthesiologists, pain nurses, and the primary care provider is replaced by the TPS [10]. The focus lies on identifying vulnerable patients at a high risk of developing CPSP, and initiating targeted interventions to prevent the progress from acute postoperative pain to CPSP. Patients with a history of preoperative opioid use, history of substance abuse, poorly controlled postoperative pain, and high postoperative opioid consumption often represent the “at-risk” group that could benefit most from enrolment into the TPS program [23•, 24]. Other patient groups at risk are those with anxiety, psychological distress, pain catastrophizing, and depression [25]. While ideally “at-risk” patients are identified in the preoperative visit and referred to the TPS team by either the surgeon or the anaesthesiologist, pathways also exist for TPS referrals in the postoperative period due to unexpected problems with acute post-surgical pain management or even after discharge due to unexpected chronic pain trajectories [11•].

### Multimodal Analgesia

Perioperative opioid sparing is a key target of TPS and can be achieved through various interventions. Postoperative analgesia is typically multimodal and includes non-opioid systemic analgesics like acetaminophen (paracetamol), non-steroidal anti-inflammatory drugs (NSAIDs), gabapentinoids (pregabalin and gabapentin), local anesthetic infusions (intravenous lidocaine), ketamine infusion, and medical cannabis [6•, 26–31]. Many of these medications can be started in the immediate preoperative period as part of “pre-emptive analgesia” to contribute to opioid sparing perioperatively [32, 33].

By targeting different receptor families along the nociceptive pathway, multimodal analgesic regimens used in TPS optimize pain control through synergistic pharmacological effects resulting in an opioid sparing effect. The resultant lower postoperative opioid requirements alleviate the incidence of opioid-related adverse side effects like nausea vomiting, constipation and respiratory depression, opioid tolerance, and opioid-induced hyperalgesia [17, 28, 34].

Intravenous lidocaine infusion has been shown to be a valuable analgesic adjunct leading to a reduction in postoperative pain and reduced use of opioids postoperatively [35]. However, the impact of lidocaine infusion in reducing the incidence of CPSP is currently unclear. A single study showed that perioperative lidocaine use during breast surgery reduced the rate of CPSP at a 3-month follow-up [29]. Intravenous ketamine has also shown to be opioid sparing in a perioperative setting in patients who have undergone surgery, particularly those who are opioid non-naïve and those with preoperative chronic pain [6•, 14•, 36]. In a systematic review and meta-analysis of 14 randomized controlled trials, the perioperative use of ketamine for 24 s following surgery was shown to reduce the incidence of CPSP at 3 months and 6 months after surgery [30]. On the other hand, studies on the role of gabapentinoids in the prevention of CPSP have been largely equivocal [6•, 30]. While the role of medical cannabis as a postoperative analgesic is under evaluation, its role in preventing CPSP is still unclear [31, 37].

Furthermore, the liberal use of loco-regional techniques including incision site infiltration, wound catheters, and nerve blocks has been encouraged within the multimodal analgesia framework [14•, 27]. Single shot or continuous nerve blocks have been shown to especially useful in extremity limb surgeries, and are recommended for acute postoperative pain management [33, 38]. Likewise, central neuraxial blocks have shown to have significant opioid sparing effects following major abdominal, thoracic procedures and intra-abdominal vascular surgeries [39, 40].

### Non-pharmacological Interventions

Non-pharmacological interventions like physiotherapy are another important foundation of a TPS [22]. Prehabilitation is the process where patients with reduced preoperative functionality are provided targeted interventions to improve their physical capabilities before major elective surgery. It includes physiotherapy, nutritional support, patient education, and specific muscle training [41]. Prehabilitation appears to improve postoperative outcomes and functional recovery resulting in shortened length of hospital stay in adults undergoing major colorectal, thoracic, and breast surgery [42–46]. Other non-pharmacological interventions like acupuncture and yoga have also been evaluated in the perioperative setting, and the early results appear promising [16••, 22, 47].

### Psychological Therapy

The psychological basis for chronic pain is well established [48]. Indeed, several psychological risk factors have been implicated in the chronification of acute postoperative pain. These include higher vulnerability to pain traumatization, pain catastrophizing, preoperative anxiety disorders, and negative affective states including depression [11•, 19]. Thus, psychological preparation and therapy is another key component of the TPS. Psychological patient preparation within the TPS program begins preoperatively with patient education regarding the potential for, consequences, and treatment modalities for acute postoperative pain, so that patients have a realistic expectation about their pain trajectories and therapy options available. Further, psychotherapy based on mindfulness, cognitive-based behavioral therapy and acceptance, and commitment therapy models are employed for surgical patients while in the hospital and post-discharge to break a vicious circle of pain and experienced distress [6•, 11•, 48]. Consequently, patients learn pain-coping mechanisms to reduce the intensity of their experienced pain and improve resilience postoperatively [49]. Therapy sessions can be held within a group setting or on an individual basis [17]. Individual personality traits, such as anxiety, depression, and pain catastrophizing, are addressed [11•].

The implementation of cognitive behavioral therapy is recommended in the postoperative pain management guidelines of the American Pain Society [33]. Several studies have shown the benefits of preoperative mindfulness-based interventions and cognitive behavioral therapy in patients with psychological risk factors for chronification of pain [23•, 27]. More recently, Acceptance and Commitment Therapy (ACT) has been explored in the perioperative setting to strengthen positive emotions and improve resilience to pain with an aim to not only reduce the intensity of pain but also improve the correlated distress and disability.

### Outpatient Care

At discharge, patients are presented with a plan for individualized pain management. Patients are then followed up for 3 to 6 months to assess their trajectory of pain and to successfully wean them off their opioid medications [4, 23•]. The continuation of follow-up care allows for the recognition of patients with an abnormal or unexpected pain trajectory, so that additional interventions can be initiated promptly. At the end of TPS care, patients are transferred back to their primary care provider. In case of ongoing pain or persistent opioid use, they are referred to a chronic pain specialist for further care and follow-up [10, 19]. The integration of mobile e-health platforms and digital applications to improve patient self-management and monitoring has shown promising preliminary results [8, 14•, 50, 51]. While preoperative quantitative sensory testing has been shown to be useful in some cohorts, its value in outpatient care and pain management after discharge remains to be evaluated [52–54].

## Evidence Regarding TPS

The utility and potential benefits of the interventions of a TPS have been investigated in several studies. In most of these studies, the main outcome measured was the reduction in postoperative opioid use in surgical patients.

In one of the first such trials from the Toronto General Hospital, Canada, Clarke et al. in 2018 studied 251 patients enrolled in the TPS program in their institution, who were either opioid naïve (*n* = 112) or opioid non-naïve (*n* = 139). Postoperative opioid consumption in terms of oral morphine milligram equivalents (MME) was reduced by 65% in the opioid naïve group and 44% in the opioid non-naïve group at 6 months post-discharge [55]. Furthermore, 45% and 26% patients in the opioid naïve and opioid non-naïve group were able to completely discontinue their opioid medication at the time of discharge from the TPS intervention [55]. More recently, in a pre-post study design, a total of 164 patients who underwent major orthopedic surgery enrolled in the TPS program were compared with a historical cohort of 172 pre-TPS patients [10]. The study investigators found a statistically significant reduction in the proportion of patients continuing opioid therapy at 90 days after surgery. Following the implementation of TPS, the percentage of patients needing opioids reduced from 27 to 13%. Under the TPS program, more opioid non-naïve users were able to wean their opioid consumption to levels below their presurgical baseline or even to discontinue their chronic opioid use (68% post-TPS vs. 45% pre-TPS) [10].

In another study by the same investigator group, an additional 49 patients with known risk factors for chronic opioid use undergoing non-orthopedic surgery were enrolled in the TPS program and analyzed along with the earlier cohort of 164 patients who underwent orthopedic surgery [27]. Of the total of 213 patients, 60 and 153 were opioid non-naïve and opioid naïve respectively. At 90 days after surgery, over 43% of chronic opioid users were able to taper their medication completely, 28% were able to reduce below their preoperative baseline, and 23% returned to their baseline with only 5% of patients remaining at higher postoperative MME. Of the 153 patients in the opioid-naïve group, only 1 patient was unable to discontinue opioids 90 days after surgery. The TPS used by this author group included a combination of preoperative patient education, perioperative multimodal analgesia with non-opioid analgesics and regional anesthesia, and psychological therapy to every patient [27].

More recently, Featherall et al. compared a TPS cohort (*n* = 137) to an historical pre-TPS control (*n* = 71) at 90 days after discharge to assess the postoperative opioid use and outcomes after total joint arthroplasty. Patients within the TPS group received preoperative education, multimodal analgesia with loco-regional interventions, and individualized psychological sessions [56]. The amount of opioid used at discharge from the hospital and at 90 days following discharge was lesser in the TPS cohort compared to the pre-TPS control group, and the difference was statistically significant. Furthermore, pain interference scores in the postoperative period decreased rapidly below the presurgical baseline in the TPS cohort, indicating a positive recovery profile [56].

In a retrospective cohort study with matched cohort pairs, Ladha et al. matched 209 TPS patients with 209 non-TPS patients who underwent surgery at other academic centers [57]. Over a follow-up period of 12 months, the mean daily opioid dose decreased by 3.53 MME/month in the TPS group compared to a decline of only 1.05 MME/month in the control group, and this difference was statistically significant [57]. Similar beneficial effects of the TPS in reducing postoperative pain and opioid consumption were demonstrated by Yu and colleagues in patients undergoing solid organ transplantation [58].

The TRUSt study was the first randomized controlled trial evaluating the effectiveness of TPS in patients undergoing surgery with an increased risk of developing CPSP and took place in a tertiary hospital in Amsterdam [20••]. A total of 176 participants were randomized into either standard care (*n* = 84) or TPS (*n* = 92) care. Unlike earlier studies, the primary outcome was the difference in quality of recovery on day 3 after surgery measured by the QoR-15 score. Secondary outcomes included intergroup differences in opioid consumption postoperatively. The study investigators found that there was no significant difference regarding the quality of recovery on day 3 following surgery. The median [IQR} reduction in total morphine milligram equivalents for standard care was 0[− 56,0] at 3 and 0[− 60,7.5] at 6 months, and TPS care was − 30[− 60,0] at 3 and − 29.3[− 65.6,0] at 6 months, respectively. Furthermore, the physical improvement was shown with lower rates of patients developing a new functional impairment in the TPS group compared to the standard care group (9.8% TPS vs. 16.7% standard care) [20••]. Subjectively, 81% of the staff in the TRUSt study found the TPS to be an advancement in patient care with 88% suggesting the program be continued [59].

## Current Lacunae in TPS and Further Direction for TPS-Related Research

While the results of TPS and its impact on postoperative opioid appear promising, there do exist several lacunae in the practice of TPS in its current form. Furthermore, these deficiencies could have an implication in interpreting the true benefits of TPS. In Table 1, we have outlined some of these problems and attempted to provide potential solutions as a roadmap for improving the impact of TPS on CPSP.

**Table 1:** Current problems with TPS and potential solutions for the future

	Current problems	Potential solutions
Outcome measurement	- Most studies focus only on postoperative opioid consumption	- Effectiveness of a TPS in reducing the incidence of CPSP needs to be demonstrated - Studies exploring the impact of TPS on other patient-centered long-term outcome measurements (ex: quality of recovery, pain intensity and pain interference scores, time to resume activities of daily living) need to be conducted
Quality of evidence	- Most studies published are observational and cohort (prospective and retrospective) - Very few RCTs currently published	- Adequately powered RCTs with robust methodology required to further research findings - Subsequent meta-analysis of RCTs would provide higher evidence base regarding the impact of TPS
E-Health and digital application Implementation	- Patient follow-up following discharge from the hospital could be challenging in certain circumstances - Patients might have barriers in accessing healthcare systems following discharge	- Integrating e-health through information technologies for the postoperative period have been used and need further exploration in the context of TPS - Digital software and mobile applications need to be explored to address the care gap after hospital discharge while providing easy to access long-term follow-up
Financial considerations	- Hospitals might have reservations regarding TPS due to the potential costs and human resources needed to run the services	- TPS could be financially possible in hospitals that are high volume centers managing major surgery - Use specific criteria to identify high-risk patients preoperatively to refer them to TPS - Targeting high-risk patients for enrollment could help to optimize the cost–benefit ratio while conserving staffing - Providing financial incentives for well performing hospitals to encourage the evidence-based practice of TPS

## Conclusion

CPSP continues to represent a significant problem for patients and healthcare delivery systems. Positioned as a key perioperative interdisciplinary link, a robust TPS could help identify “at-risk” patients and provide targeted evidence-based interventions to prevent the transition of poorly controlled acute postoperative pain to CPSP. Studies have shown that patients who have received TPS care have significant reductions in their postoperative long-term opioid use with better rates of opioid weaning and discontinuation. Functional outcomes, pain severity, and pain interference also appear to be improved. Further adequately powered randomized control trials are needed to look into the potential benefits of TPS beyond a mere reduction in postoperative opioids. The potential impact of TPS in improving patient-reported outcomes and long-term functional outcomes needs to be further explored.

## Data Availability

No datasets were generated or analysed during the current study.
